# Expression patterns of CEACAM5 and CEACAM6 in primary and metastatic cancers

**DOI:** 10.1186/1471-2407-7-2

**Published:** 2007-01-03

**Authors:** Rosalyn D Blumenthal, Evelyn Leon, Hans J Hansen, David M Goldenberg

**Affiliations:** 1 Garden State Cancer Center, Center for Molecular Medicine and Immunology, Belleville, NJ 07109, USA (RDB, EL, DMG), and Immunomedics, Inc., Morris Plains, NJ 07950, USA (HJH)

## Abstract

**Background:**

Many breast, pancreatic, colonic and non-small-cell lung carcinoma lines express CEACAM6 (NCA-90) and CEACAM5 (carcinoembryonic antigen, CEA), and antibodies to both can affect tumor cell growth in vitro and in vivo. Here, we compare both antigens as a function of histological phenotype in breast, pancreatic, lung, ovarian, and prostatic cancers, including patient-matched normal, primary tumor, and metastatic breast and colonic cancer specimens.

**Methods:**

Antigen expression was determined by immunohistochemistry (IHC) using tissue microarrays with MN-15 and MN-3 antibodies targeting the A1B1- and N-domains of CEACAM6, respectively, and the MN-14 antibody targeting the A3B3 domain of CEACAM5. IHC was performed using avidin-biotin-diaminobenzide staining. The average score ± SD (0 = negative/8 = highest) for each histotype was recorded.

**Results:**

For all tumors, the amount of CEACAM6 expressed was greater than that of CEACAM5, and reflected tumor histotype. In breast tumors, CEACAM6 was highest in papillary > infiltrating ductal > lobular > phyllodes; in pancreatic tumors, moderately-differentiated > well-differentiated > poorly-differentiated tumors; mucinous ovarian adenocarcinomas had almost 3-fold more CEACAM6 than serous ovarian adenocarcinomas; lung adenocarcinomas > squamous tumors; and liver metastases of colonic carcinoma > primary tumors = lymph nodes metastases > normal intestine. However, CEACAM6 expression was similar in prostate cancer and normal tissues. The amount of CEACAM6 in metastatic colon tumors found in liver was higher than in many primary colon tumors. In contrast, CEACAM6 immunostaining of lymph node metastases from breast, colon, or lung tumors was similar to the primary tumor.

**Conclusion:**

CEACAM6 expression is elevated in many solid tumors, but variable as a function of histotype. Based on previous work demonstrating a role for CEACAM6 in tumor cell migration, invasion and adhesion, and formation of distant metastases (Blumenthal et al., Cancer Res 65: 8809–8817, 2005), it may be a promising target for antibody-based therapy.

## Background

The human carcinoembryonic antigen (CEA) family has 7 genes belonging to the CEACAM subgroup. These subgroup members are mainly associated with the cell membrane and show a complex expression pattern in normal and cancerous tissues. The CEACAM5 gene, also known as CD66e, codes for the protein, CEA [1,2]. CEACAM5 was first described in 1965 as a gastrointestinal oncofetal antigen [3], but is now known to be overexpressed in a majority of carcinomas, including those of the gastrointestinal tract, the respiratory and genitourinary systems, and breast cancer [4-8]. CEACAM6 (also called CD66c or NCA-90) is a non-specific cross-reacting glycoprotein antigen that shares some antigenic determinants with CEACAM5 [9]. CEACAM6 also is expressed on granulocytes and epithelia from various organs, and has a broader expression zone in proliferating cells of hyperplastic colonic polyps and adenomas, compared with normal mucosa [10], as well as by many human cancers [10-12]. Relatively high serum levels of CEACAM6 are found in patients with lung, pancreatic, breast, colorectal, and hepatocellular carcinomas. The amount of CEACAM6 does not correlate with the amount of CEACAM5 expressed [11].

Expression of CEACAM6 in colorectal cancer correlates inversely with cellular differentiation [13] and is an independent prognostic factor associated with a higher risk of relapse [14]. Both CEACAM5 and CEACAM6 have a role in cell adhesion, invasion and metastasis. CEACAM5 has been shown to be involved in both homophilic (CEA to CEA) and heterophilic (CEA binding to non-CEA molecules) interactions [15-17], suggesting to some that it is an intercellular adhesion molecule involved in cancer invasion and metastasis [18-20]. These reactions were completely inhibited by the Fab' fragment of an anti-CEACAM5 antibody [16]. CEACAM6 also exhibits homotypic binding with other members of the CEA family and heterotypic interactions with integrin receptors [17]. Antibodies that target the N-domain of CEACAM6 interfere with cell-cell interactions [21]. We have reported previously that many breast, pancreatic, colonic and non-small-cell lung cancer (NSCLC) cell lines express CEACAM6, and that anti-CEACAM6 antibody inhibits in vitro migration, invasion, and adhesion of antigen-positive cells [22]. Therefore, the ability to interfere with CEACAM6-mediated homotypic and heterotypic binding might have beneficial anti-metastatic effects.

The goals of the current study were to: (1) use tissue microarray analysis to compare the relative expression of CEACAM5 and CEACAM6 in different histotypes of solid tumors, and (2) develop additional supportive evidence for a role for CEACAM6 in metastasis by comparing expression between primary sites and matched metastases in the same patients. This is the first such comparison of these two CEACAM antigens in such matched patient specimens.

## Methods

### Antibodies

MN-15 binds to the A1B1-domain (Gold group 4) and MN-3 [22] binds to the N-domain (Gold group 5) found on both CEACAM5 and CEACAM6 [23]. MN-14 binds to the A3B3 domain (Gold group 3) only found on CEACAM5 [24]. These antibodies have similar affinities for their target antigens [25]. These antibodies, and the non-specific Ag8 IgG, were supplied by Immunomedics, Inc. (Morris Plains, NJ). MN-3 and MN-15 were used as murine MAbs, while MN-14 was included in its humanized form, hMN-14 or labetuzumab [26].

### Tissue microarrays

AccuMax tissue arrays were purchased from ISUABXIS through Accurate Chemical & Scientific Corp (Westbury, NY). The following arrays were used: Breast A202 (II), colon with matching liver metastases A203 (II), lung A206, pancreatic (A207), prostate A208, and ovary A213. Additional breast (BR1001), colorectal (C0991), and lung (LC810) arrays of matching primary tumor and lymph node metastases were purchased from US Biomax, Inc. (Rockville, MD). All arrays consisted of duplicate cancer tissue cores of varying histotypes and four non-neoplastic corresponding samples on each slide. There were 45 breast, 40 lung, 26 pancreatic, 40 prostate, and 45 ovarian cancer specimens (Table 1). Some histotypes are well represented (e.g., 30 infiltrating ductal breast tumors, while others have only 3–6 cores per histotype. The metastasis arrays consisted of the following matched cases: 18 normal colon, primary colon cancer and liver metastases, 38 breast and lymph node metastases, 33 colon and lymph node metastases, and 37 lung and lymph node metastases.

**Table 1:** Number of Tissue Cores Analyzed for Each Histotype.

**Tumor**	**Histotype**	**N**	**Tumor**	**Histotype**	**N**
**Breast**	Infiltrating Ductal	30	**Colon**	Adenocarcinoma	41
	Papillary	8	**Pancreas**	Well Diff	1
	Lobular	4		Well-Mod Diff	16
	Phyllodes	4		Mod Diff	2
**Lung**	Well Diff Adeno	5		Mod-Poorly Diff	6
	Mod Diff Adeno	5		Poorly Diff	1
	Poorly Diff Adeno	5	**Ovary**	Serous Adeno	5
	Well Diff Squamous	5		Mucinous Adeno	4
	Mod Diff Squamous	5		Clear Cell	5
	Poorly Diif Squamous	5		Transitional Cell	5
	Bronchioalveolar	3		Endometroid	4
	Large-Cell Neuroendocrine	2		Brenner	4
	Large-Cell	3		Yolk Sac	3
	Small-Cell	2		Granulosa	3
**Prostate**	Stage II	21		Dysgerminoma	3
	Stage III	15			
	Stage IV	4			

### Immunohistochemistry

Slides were deparaffinized in xylene, rehydrated, and treated with fresh 0.3% hydrogen peroxide in methanol for 15 min. Following a wash in 1× phosphate-buffered saline (PBS, pH 7.4), slides were blocked with normal serum in a humid chamber for 20 min at room temperature (RT). Excess serum was rinsed off with 1× PBS and slides were incubated in a humid chamber with 25–50 μl of primary antibody (10 μg/ml) for 45 min at RT. For CEACAM5 staining, the primary antibody was murine mMN-14 IgG. For CEACAM6 staining, slides were first blocked with humanized hMN-14 IgG and then incubated with primary antibody, either murine mMN-15 or mMN-3 IgG. Excess primary antibody was washed off and sections were covered with biotinylated goat-anti-mouse (Vectastain ABC kit) for 30 min in humid chamber at RT. Slides were then flooded with 0.3% H_2_O_2 _in methanol and 25 μl avidin-horseradish peroxidase (HRP) conjugate (ABC) was added. Slides were incubated for 45 min at RT, washed in 1× PBS, and covered with 100 μl 3,3'-diaminobenzidine tetrahydrochloride solution (100 mg/ml diaminobenzide in 0.1 M sodium acetate buffer, pH 6.0, with 0.01% (v/v) H_2_O_2_) for 15 min. Slides were washed twice by dipping in tap water and counterstained with 4 quick dips in hematoxylin (filtered through Whatman #4 filter paper). Slides were rinsed, air-dried, and mounted with 1–2 drops of cytoseal and a glass coverslip. The method of Kawai was used to calculate a semi-quantitative score from 0 to 8 for staining of each tissue core. The number of positive cells/filed was estimated and assigned a number: 0 = none, 1 = 1/100 cells, 2 = 1/100 to 1/10 cells, 3 = 1/10 to 1/3 cells, 4 = 1/3 to 2/3 cells, and 5 = >2/3 cells. The intensity of staining was then determined where 0 = none, 1 = weak, 2 = intermediate, and 3 = strong. The first and second scores were then added together resulting in a maximum staining score of 8 for any tissue core [27]. Two independent blinded investigators (author 1 and 2) performed IHC analysis and results were strongly consistent between the two readings. Results were recorded as the mean ± standard deviation for each group. Comparisons between CEACM5 and CEACAM6 scores for a given histotype or between histotypes for each antigen were assessed by a one-factor analysis of variance with the use of a two-tailed F test and a 95% confidence limit. The null hypothesis Ho: μ1 = μ2 = 1/4 μ*k*, where *k *equals the number of experimental groups, was used. A two-tailed test takes into account an extreme value in any one group that deviates from the population mean in either the high or low direction (two-sided). The F value is a measure of the probability that this difference in groups could occur by chance alone.

## Results

### Expression in solid tumors as a function of histotype

**Figure 1:**
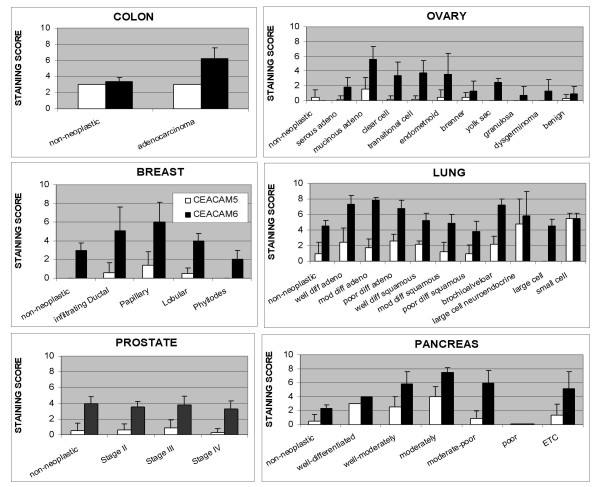
CEACAM5 and CEACAM6 staining of colon, ovarian, breast, lung, prostate and pancreatic tissue core specimens as a function of tumor histotype is summarized. The results graphed represent the mean ± standard deviation for each histotype and antigen.

For all tumor cores evaluated, the amount of CEACAM6 was greater than that of CEACAM5. However, the homogeneity of expression and staining intensity varied between tissue histotypes and between samples within the same histological type. A summary of staining scores for CEACAM5 and CEACAM6 for each tumor type and histological type is presented in Figure 1.

**Figure 2:**
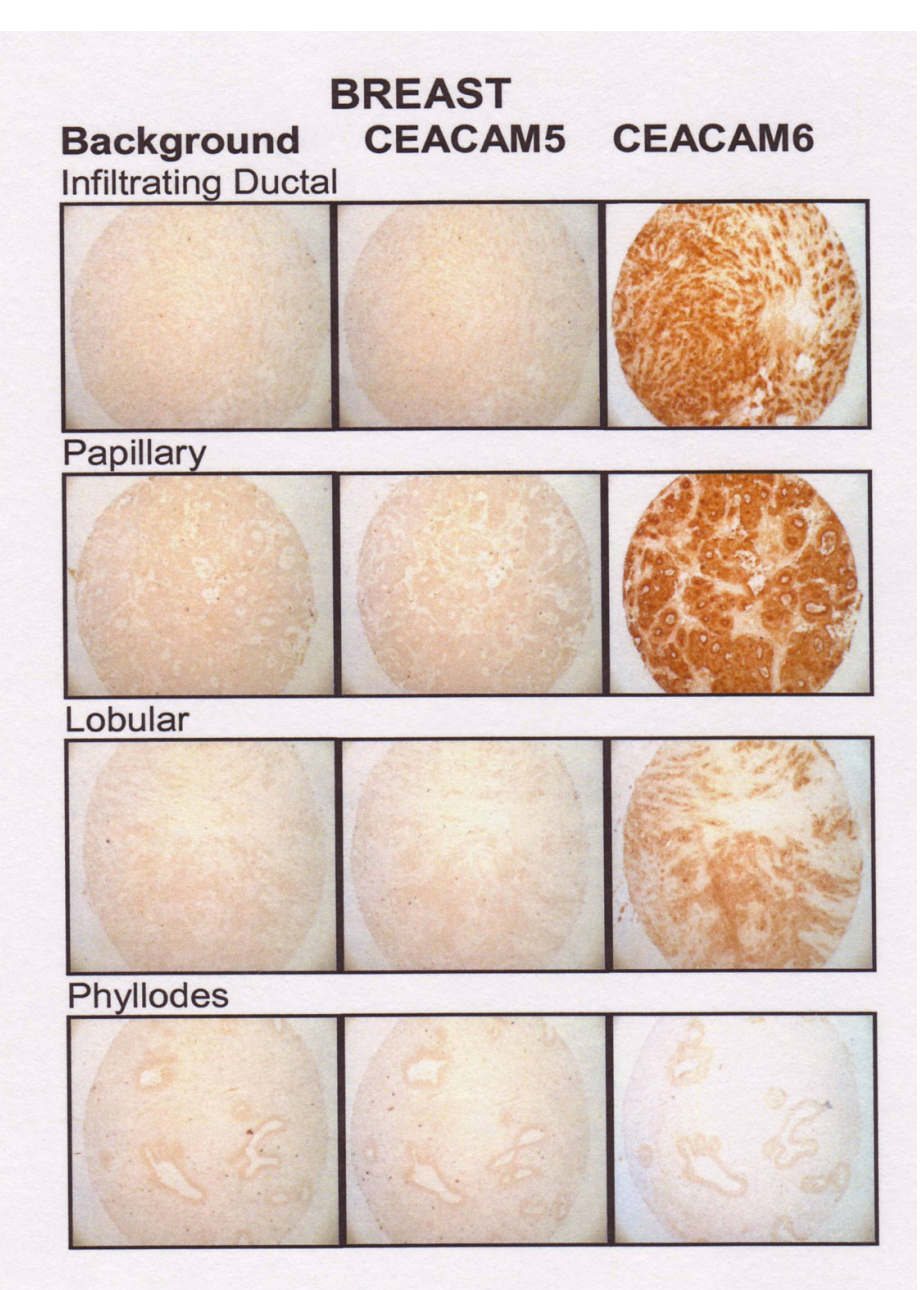
CEACAM5 and CEACAM6 expression in representative cases of infiltrating ductal, papillary, lobular and phyllodes breast tumor cores.

We evaluated 45 breast tumor cores: 30 infiltrating ductal carcinoma, 8 papillary, 4 lobular, and 3 phyllodes. CEACAM6 levels were higher than CEACAM5 levels for all histotypes (P < 0.001). The highest CEACAM6 expression was found in papillary (6.0 ± 2.1) > infiltrating ductal (5.1 ± 2.5) > lobular (4.0 ± 0.8) > phyllodes (2.0 ± 1.0). The differences between papillary and lobular breast cancers were significant at the P < 0.01 level. The highest CEACAM5 expression was found in papillary samples (1.4 ± 1.4), but was not statistically different from infiltrating ductal or lobular samples. Pyllodes breast cancer is a stromal tumor, usually benign, and should therefore not express CEACAM5 or CEACAM6. Examples of CEACAM5 and CEACAM6 staining for each histotype are found in Figure 2.

**Figure 3:**
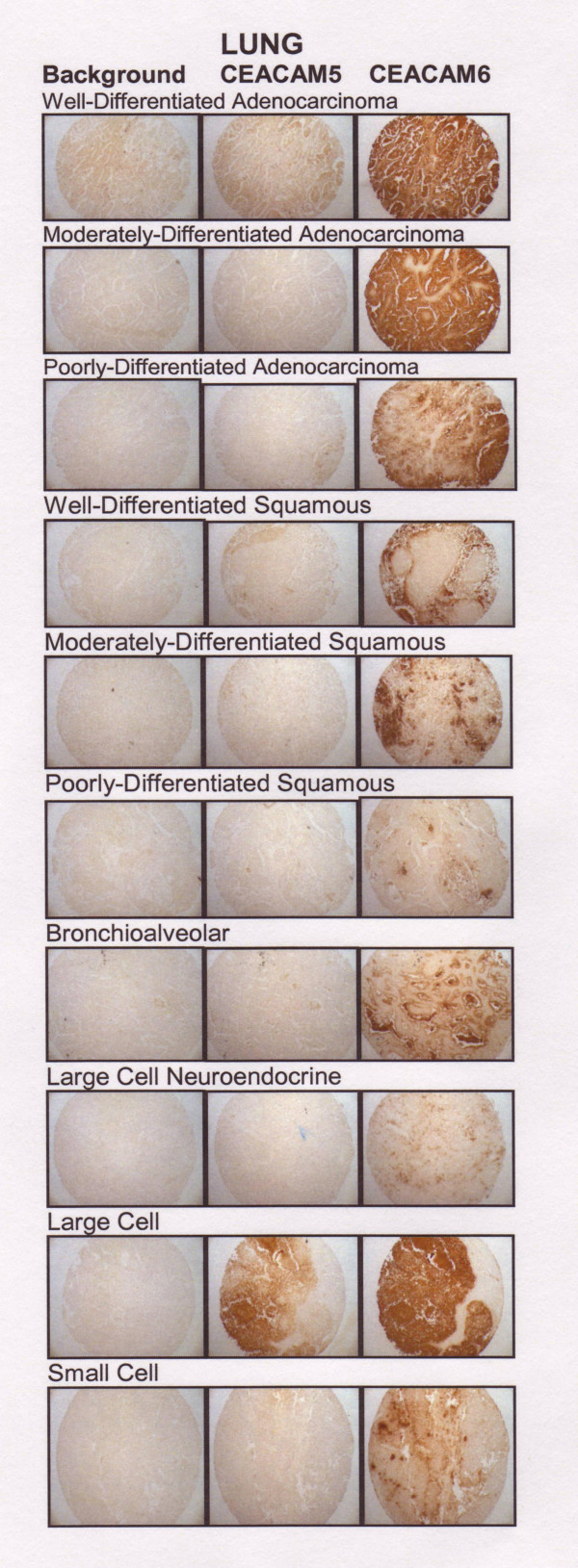
CEACAM5 and CEACAM6 expression in representative cases of well-, moderately-, poorly differentiated adenocarcinoma, squamous carcinoma of lung, brochioalveolar, large-cell neuroendocrine, large-cell, and small-cell lung carcinoma cores.

CEACAM5 and CEACAM6 expression was assessed in 6 different lung cancers: 5 each of well, moderately and poorly differentiated adenocarcinoma, 5 each of well, moderately and poorly differentiated squamous carcinoma, 3 each of large cell and bronchioalveolar, and 2 each of large cell neuroendocrine and small cell cancer. Among these, adenocarcinoma expressed more CEACAM6 than squamous cancer (P < 0.001). The highest CEACAM6 expression was found in moderately-differentiated adenocarcinoma (7.8 ± 0.4) > well-differentiated adenocarcinoma (7.3 ± 1.1) = bronchioalveolar (7.2 ± 0.8) > poorly-differentiated adenocarcinoma (6.8 ± 1.0) > small-cell (5.5 ± 0.7) > well-differentiated squamous (5.2 ± 1.0) > moderately-differentiated squamous cancer (4.9 ± 1.1). CEACAM6 levels in large-cell (4.5 ± 0.9) and poorly-differentiated squamous carcinomas (3.8 ± 1.3) were similar to non-neoplastic lung tissue (P = NS), suggesting that anti-CEACAM6 antibodies would not be effective with these histotypes of lung cancer. The highest expression of CEACAM5 was in small-cell lung cancer specimens (5.5 ± 0.7), followed by large-cell neuroendocrine tumors (4.75 ± 3.18). Large-cell tumors were CEA-negative and all adenocarcinomas and serous tumors scored ≤ 2.60. Typical examples of CEACAM5 and CEACAM6 staining for each histological type are shown in Figure 3.

**Figure 4:**
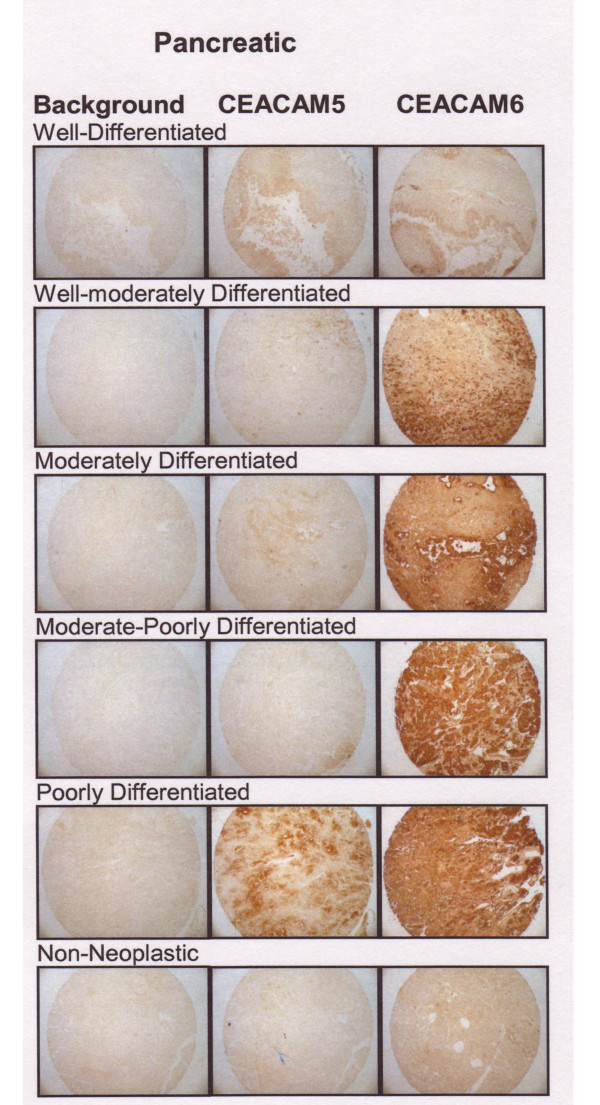
CEACAM5 and CEACAM6 expression in representative cases of well-, moderately-well-, moderately-, moderately-poorly, and poorly-differentiated adenocarcinoma and non-neoplastic pancreatic tissue cores.

Pancreatic cancer has been the most extensively studied neoplasm with respect to CEACAM6 expression [28-35]. In this work, we evaluated CEACAM5 and CEACAM6 as a function of tumor cell differentiation. One well-differentiated, 3 well-moderately differentiated, 13 moderately-differentiated, 2 moderately- to poorly-differentiated, and 7 poorly-differentiated tumor cores were studied. The highest expression of CEACAM6 in pancreatic tumors was found in moderately- (7.5 ± 0.7) > moderately-poor (5.9 ± 1.9) = well-moderately differentiated (5.8 ± 1.8) > poorly-differentiated tumors (5.1 ± 2.5) > well-differentiated (4.0 ± 0.0) adenocarcinomas (P = NS between the subtypes). Non-neoplastic pancreas CEACAM6 expression was 2.25 ± 0.5. The well-moderately, moderately, and moderately-poor adenocarcinomas were significantly higher than non-neoplastic pancreas (P < 0.001). CEACAM6 expression did not correlate with disease stage. Samples with high (8) and low (3–4) expression could be found in stages IA-IB, IIA-IIB, and IV. CEACAM5 expression was lower than CEACAM6 for all histotypes; the highest expression being found in moderately differentiated tumors (4.0 ± 1.4) and the least in the moderate-poor (0.92 ± 1.92) and poorly-differentiated (1.4 ± 1.5) tumors. Only the moderately and the well-moderately differentiated tumors expressed significantly more CEACAM5 than non-neoplastic tissues (P < 0.002 and P < 0.005, respectively). Examples of CEACAM5 and CEACAM6 staining for each histotype are presented in Figure 4.

**Figure 5:**
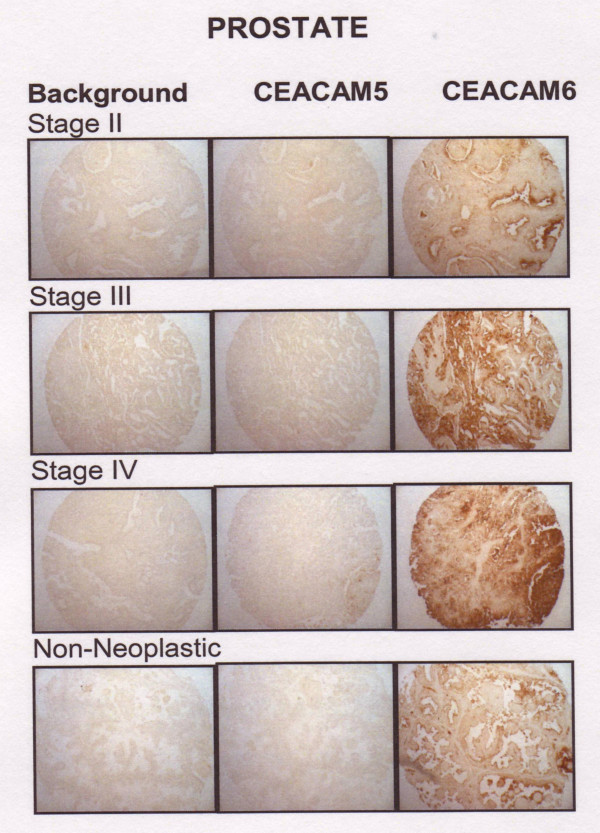
CEACAM5 and CEACAM6 expression in representative stage II, stage III, and stage IV prostate cancer cases with non-neoplastic prostate tissues.

Eighteen stage-II, 15 stage-III, and 4 stage-IV prostate tumor cores were stained for CEACAM5 and CEACAM6. Gleason scores of 4 to 9 were represented in the stage-II samples, and Gleason scores of 6 to 10 were found in the stage-III specimens. All stage-IV samples were Gleason 9–10. Expression did not correlate with Gleason score of the sample within any stage. Similar expression of CEACAM6 was found in stage-II, -III, and -IV prostate cancer (3.3–3.8), and was not significantly different from non-neoplastic prostate tissue (P = NS). CEACAM5 expression was consistently below 0.9 for all stages of prostate cancer and was not greater than expression levels in non-neoplastic prostate tissue (0.5 ± 1.0; P = NS), suggesting that prostate tumors would not be responsive to treatment with either anti-CEACAM5 or anti-CEACAM6 antibody therapies. Examples of CEACAM5 and CEACAM6 staining for each histological type are shown in Figure 5.

**Figure 6:**
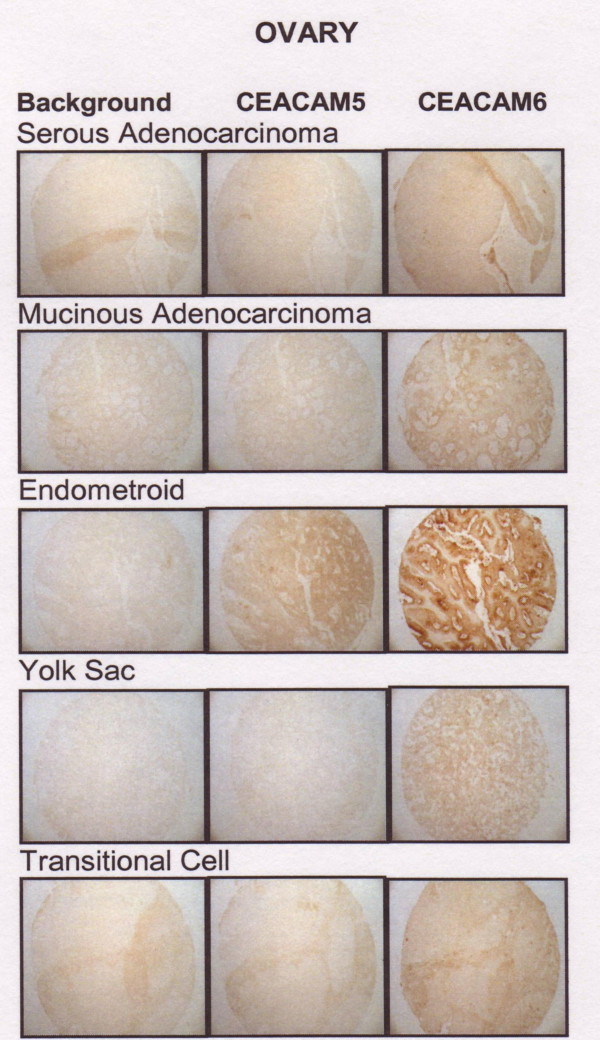
CEACAM5 and CEACAM6 expression in representative non-neoplastic and adenocarcinoma cases of the colon.

Nine ovarian cancer types were included in these studied: 5 each of serous adenocarcinoma, mucinous adenocarcinoma, clear cell carcinoma, and transitional cell carcinoma; 4 each of endometrioid adenocarcinoma and Brenner tumor; and 3 each of yolk sac tumor, granulosa cell tumor, and dysgerminoma. The amount of CEACAM6 in ovarian cancer was highest in mucinous adenocarcinoma (5.6 ± 1.7) > transitional cell (3.8 ± 1.6) > endometrioid (3.6 ± 2.8) > clear cell (3.4 ± 1.8) > yolk sac tumors (2.5 ± 0.5). Mucinous tumor CEACAM6 expression was significantly higher than transitional and endometroid (P < 0.02), clear cell (P < 0.01), and yolk sac tumors (P < 0.005). Much lower levels were found in serous adenocarcinoma (1.8 ± 1.3) > Brenner tumor (1.3 ± 1.4) = dygerminoma (1.3 ± 1.5) > granulosa cell (0.7 ± 1.2). Normal ovary samples were negative for CEACAM6. Thus, all tumor histotypes expressed significantly more CEACAM6 than non-neoplastic ovary. The highest CEACAM5 expression also was found in the mucinous adenocarcinoma type (1.6 ± 1.5). Expression of CEACAM5 in all other ovarian samples scored below 0.6, and non-neoplastic ovary scores were 0.5 ± 1.0. Mucinous CEACAM5 levels were significantly higher than all other histotypes (P < 0.002 compared with endometroid and Brenner tumors, and P < 0.001 compared with serous, clear cell, transitional and yolk sac). Examples of CEACAM5 and CEACAM6 staining for each histotype are found in Figure 6.

**Figure 7:**
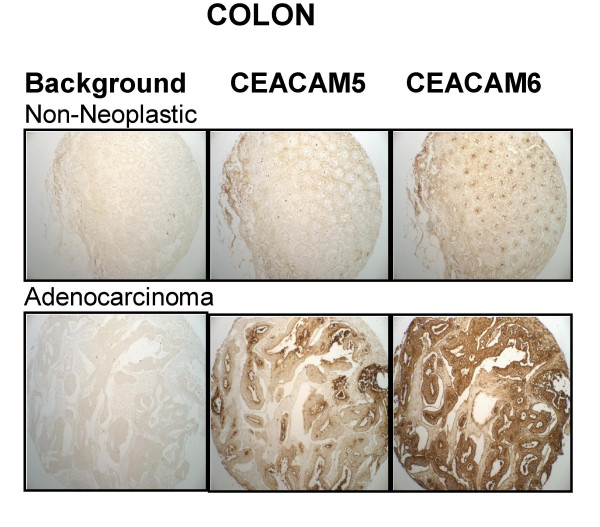
CEACAM5 and CEACAM6 expression in representative cases of serous and mucinous adenocarcinoma, endometroid, yolk sac, and transitional cell carcinoma of the ovary.

Much larger amounts of CEACAM6 were found in colon adenocarcinoma (6.2 ± 1.4) compared with non-neoplastic colon (3.0 ± 0.0; P < 0.002) and CEACAM6 expression exceeded CEACAM5 expression (3.4 ± 0.5; P < 0.001). Examples of CEACAM5 and CEACAM6 staining for each histotype are found in Figure 7.

**Figure 8:**
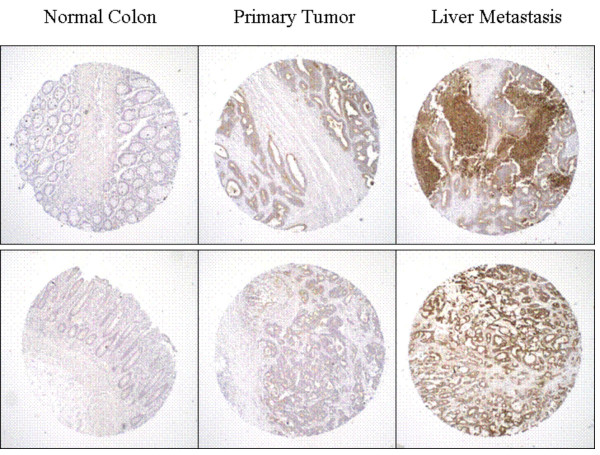
Immunohistochemical staining of four matched patient specimens from normal colon, primary colon carcinoma, and liver metastasis stained with MN-15 anti-CEACAM6.

CEACAM6 expression has been associated with cell adhesion, a key step in the metastatic cascade. We have shown that antibody to CEACAM6 expression can block adhesion [22]. Therefore, we assessed whether CEACAM6 expression was similar or different between matched primary colon and metastatic liver sites. In half of the matched cases (N = 6), CEACAM6 expression was much greater in the liver metastasis than in the primary colon tumors, and in the remaining 6 cases, the amounts were comparable between the primary and the metastatic liver sites. Two examples of CEACAM6 staining for matched normal colon tissue, primary colon tumor, and liver metastases are shown in Figure 8.

In contrast to the higher expression off CEACAM6 in many secondary liver sites from colon cancer, there was no pattern for CEACAM6 expression between primary tumor and lymph node metastases. For breast samples, the lymph node sites had higher CEACAM6 expression in 7 pairs, lower CEACAM6 in 6, and no difference in 25 pairs. For lung samples, the lymph node sites had higher CEACAM6 expression in 10 pairs, lower CEACAM6 in 11, and no difference in 16 pairs. For colon samples, the lymph node sites had higher CEACAM6 expression in 7 pairs, lower CEACAM6 in 10, and no difference in 11 pairs (Table 2).

**Table 2:** Comparison of CEACAM6 Expression between Primary and Lymph Node Metastases in Patients with Matched Specimens.

	**Number of Cases Where Primary Tissue Expression was Higher than Lymph Node Expression**	**Number of Cases Where Primary Tissue Expression was Lower than Lymph Node Expression**	**Number of Cases Where Primary Tissue and Lymph Node Expression was Similar**
**Breast**	6	7	25
**Lung**	11	10	16
**Colon**	10	7	11

## Discussion

CEACAM5 and CEACAM6 are two tumor-associated antigens that play important regulatory roles in cell adhesion and in tumor cell chemosensitivity [36-38]. CEACAM6 overexpression independently predicts poor overall survival and poor disease-free survival, whereas CEACAM5 has not been related significantly to these outcomes [39].

Studies have shown that CEACAM5 affects expression of various groups of cancer-related genes, especially cell cycle and apoptotic genes, protecting colonic tumor cells from various apoptotic stimuli, such as treatment with 5-fluorouracil [40]. Therefore, CEACAM5 expression may be a means for cancer cells to overcome apoptosis-inducing therapies. Ordonez et al. have reported that expression of both CEACAM5 and CEACAM6 plays a role in inhibiting apoptosis of cells when deprived of their anchorage to the extracellular matrix, a process known as anoikis [41]. Increased expression of CEACAM6 correlates with a decrease in sensitivity to drugs, like gemcitabine [30]. Targeting CEACAM5 and/or CEACAM6 may therefore be a novel method of modulating cancer cell chemosensitivity and apoptosis. It has been reported that siRNA to CEACAM6 impairs resistance to anoikis and increases caspase-mediated apoptosis of xenografted tumors [31]. Antibody-directed targeting of CEACAM6 may provide a clinically feasible alternative to RNA interference silencing to enhance responsive to chemotherapeutic agents in those tumors that express CEACAM6.

To determine which solid tumors and histological types would be most amenable to antibody blocking of CEACAM5 and CEACAM6, we studied expression of these antigens using tissue microarray analysis. To date, pancreatic and colonic cancer have been the focus of CEACAM6 expression in the literature [35,42]. Here, we have further explored the expression of CEACAM6 in a panel of solid tumors: breast, lung, ovary and prostate cancer, in addition to expanding on pancreatic and colonic tumors, and used tissue microarrays to further define tumors that are CEACAM6+ as a function of histological type in all six solid tumor categories. Our results show that expression is strongly dependent on the histotype of the tumor. Antigen expression in some subtypes is 2–4-fold higher than in normal tissues, while in others, expression is similar to non-neoplastic tissues. Other investigators have reported differences in the expression of select tumor antigens as a function of histotype, e.g., TAG-72 in lung cancer [43], VEGF in skin cancer [44], and BER EP4 and CA-125 in ovarian cancer [45]. However, the results in this study are the first to explore differences in both CEACAM5 and CEACAM6 as a function of tumor histotype across six tumor tissues.

The demonstration of higher CEACAM6 expression compared with CEACAM5 across most solid tumors, and the differential expression as a function of histotype, are important observations for translating anti-CEACAM6 therapy to patients. However, we appreciate that additional supportive evidence from Western blots, RT-PCR/Northern blots is needed. This semi-quantitative analysis is intended only as an initial step towards elucidating the importance of CEACAM6 as a tumor target in a variety of solid tumors that extend the many important studies reported for pancreatic cancer [28-35]. It also reveals that expression level varies as a function of tumor histotype. Since some histotypes only had 3–6 core samples and considerable variability in antigen expression within the histotype was noted, it is appropriate to include additional core tissues and to provide more quantitative support with other techniques on biopsy tissues in future studies.

We have also addressed the expression pattern of CEACAM6 in primary tumors and in matched metastases in the same patients. Our results show that in half of the clinical specimens, liver metastases had a much higher expression of CEACAM6 than the primary colorectal tumors, suggesting that in such patients, blocking adhesion and invasion that results from CEACAM6 expression might have influenced the ability of tumor cells to metastasize, as we have in fact shown experimentally [4]. However, CEACAM6 expression in lymph node metastases was similar to the amount of antigen in primary breast, colon or lung tumor samples. The mechanism by which malignant tumors invade lymphatics and metastasize to regional lymph nodes appears to be regulated by VEGF-C and VEGF-D induced lymphogenesis [46] and a chemokine gradient. Directional movement is related to chemokine receptor expression on tumor cells [47], but does not involve members of the CEACAM family. In contrast, CEACAM6 plays an important role in migration, invasion and adhesion [31,34], steps that are important in the metastatic spread to secondary tissue sites other than lymph nodes [48]. In fact, anti-adhesive molecules that disrupt cell-matrix and cell-cell attachments have been proposed as potential cancer therapeutics based on their ability to interfere with motility, adhesion, and metastatic progression [22,36,49].

We have recently reported that the humanized anti-CEA (CEACAM5) antibody, MN-14, can enhance the therapeutic effects of two cytotoxic drugs used frequently in colorectal cancer therapy, fluorouracil and CPT-11, in both subcutaneous and metastatic human colonic tumor cells propagated in nude mice [50]. In another high CEA-expressing human medullary thyroid cancer xenograft, we have also shown that MN-14 anti-CEA IgG can inhibit tumor cell growth and also augment the effects of dacarbazine, a drug that is active in this cancer type [51]. One explanation may involve a role in antibody blocking adhesion [38] and thereby chemosensitizing the tumor cells.

In a series of provocative studies, Duxbury and associates have shown that silencing CEACAM6 by siRNA: (a) enhances cell anoikis, (b) increases caspase activation in response to anchorage independent conditions, (c) downregulates the Akt cell survival pathway, (d) inhibits metastasis in vivo, and (e) enhances gemcitabine induced chemosensitivity [30,31,33-35]. Thus, in addition to CEACAM5, CEACAM6 may also represent a useful therapeutic target. Blocking CEACAM6-mediated homotypic and/or heterotypic adhesion may have anti-metastatic and chemosensitizing effects. In ongoing preclinical therapy studies, we are examining the therapeutic effects of unconjugated anti-CEACAM6 antibody alone or combined with standard chemotherapeutic agents in colon, breast, and lung metastasis models. An alternative approach is to develop an anti-CEACAM6 immunoconjugate as a therapeutic agent for CEACAM6+ tumors, as described by Duxbury et al. [32]. In vitro targeting with an anti-CEACAM6 antibody, followed by secondary saporin-conjugated immunoglobulin (IgG), induced marked cytotoxicity via caspase-mediated apoptosis. In an in vivo nude mouse xenograft model, this indirect immunotoxin approach markedly suppressed pancreatic adenocarcinoma tumor growth and enhanced tumor apoptosis.

## Conclusion

Based on expression level, CEACAM6 may be a more promising target for antibody-based anti-metastatic and chemosensitizing therapy than CEACAM5 in all the solid tumors studied. Furthermore, CEACAM6 may be a useful antigen to target in select subtypes of solid tumors, with the exception of prostate cancer, where no differentiation was seen, compared to normal prostate. In colonic cancer, CEACAM6 may play an important role in the development of distant metastases.

## Abbreviations

**CEA**, carcinoembryonic antigen; **DAB**, diaminobenzide; **IHC**, immunohistochemistry; **NCA-90**, non-specific cross-reacting antigen-90

## Competing interests

Drs Goldenberg and Hansen have management roles and financial interest (stock) in Immunomedics, which owns the antibodies tested in this study. All other authors declare that they have no potential competing interests.

## Authors' contributions

RDB conceived and designed the studies, analyzed the data and drafted the manuscript. EL performed all immunohistochemistry. HH was involved in revising the manuscript and influencing critical intellectual content. DMG was involved in data interpretation and presentation, revising the manuscript, and influencing critical intellectual content. All authors read and approved the final manuscript.

## Pre-publication history

The pre-publication history for this paper can be accessed here:


